# Supplementary material for: Complexome profiling of the Chlamydomonas psb28 mutant reveals TEF5 as an early PSII assembly factor
Source: Plant Cell. 2025 Mar 17;37(6):koaf055. doi: 10.1093/plcell/koaf055 (PMC12164590; doi:10.1093/plcell/koaf055)
Supplement: koaf055_Supplementary_Data [file koaf055_supplementary_data.zip › psb28_interactive.zip!/plots/intensity/qualityPlots/peptides/COX12_Cre06.g304350.t1.2.html]

### Cre06.g304350.t1.2

Trivial name: COX12  
SeqCoverage [median]: 25.70  
Mass: 17.25 kDa  
  
 

| Sequence | MissedCleavage | Mass [Da] | wtIntensity | muIntensity | Score |
| --- | --- | --- | --- | --- | --- |
| FNEYYK | 0 | 862.39 | 3524.84 | 10734.49 | 148.28 |
| GLWTGKY | 1 | 823.42 | 2144.78 | 0.00 | 97.60 |
| IAGEDGTK | 0 | 789.39 | 5345.84 | 7518.09 | 116.73 |
| IAGEDGTKLADFLEPELITAPYDPR | 1 | 2730.38 | 1254.40 | 0.00 | 60.94 |
| LADFLEPELITAPYDPR | 0 | 1959.00 | 14988.95 | 6092.11 | 188.91 |
|  |  |  | 27258.8 | 24344.7 |  |
